# The Importance of Dose, Frequency and Duration of Vitamin D Supplementation for Plasma 25-Hydroxyvitamin D

**DOI:** 10.3390/nu5104067

**Published:** 2013-10-11

**Authors:** Yi-Sheng Chao, Ludovic Brunel, Peter Faris, Paul J. Veugelers

**Affiliations:** 1 School of Public Health, University of Alberta, 3-50 University Terrace, 8303–112 St, Edmonton, AB T6G 2T4, Canada; E-Mail: paulus@ualberta.ca; 2 Santessence, 905 1st Ave NE Calgary, AB T2E 0C5, Canada; E-Mail: ludovic.brunel@gmail.com; 3 Alberta Bone and Joint Health Institute, Alberta Health Services, 3280 Hospital Dr NW #400 Calgary, AB T2N 4Z6, Canada; E-Mail: peter.faris@albertahealthservices.ca

**Keywords:** vitamin D, dose response, frequency, duration, 25-hydroxyvitamin D (25(OH)D)

## Abstract

The importance of dose, frequency and duration of vitamin D supplementation for plasma 25(OH)D levels is not well described and rarely reported for supplementation that exceeds 2000 IU per day. The objective is to examine dose, frequency and duration of supplementation in relation to plasma 25(OH)D in a large population-based sample. We accessed data on 2714 volunteers that contributed to 4224 visits and applied multilevel regression. Compared to not using supplements, a minimum regimen of 1000–2000 IU once or twice per week for one month was not effective in raising 25(OH)D. Compared to this minimum regimen, higher doses of 2000–3000, 3000–4000, and 5000 IU or more were associated with a 7.49, 13.19 and 30.22 nmol/L 25(OH)D increase, respectively; frequencies of three to four, five to six and seven times/week were associated with a 5.44, 16.52 and 30.69 nmol/L increase, respectively; and supplementation of five months or longer was associated with an increase of 6.68 nmol/L (*p* < 0.01 for all). Age, body weight, physical activity, smoking, and self-rated health were significantly associated with 25(OH)D. Whereas dose, frequency and duration of supplementation are important to healthy subjects committed to optimizing their nutritional status, to the design of clinical trials, individual characteristics and lifestyle contribute substantially to 25(OH)D.

## 1. Introduction

Vitamin D plays an important role in maintaining bone health and has benefits for extra-skeletal health [1]. Daily intake of 600 IU of vitamin D [2] and other dosages have been recommended [3]. However, vitamin D intake is not an effective measure of vitamin D status in human bodies. Plasma 25-hydroxy-vitamin D [25(OH)D] levels are the established proxy to assess health benefits [4,5].

The dose response relationship between supplementation and plasma 25(OH)D is the key to understanding the intervention effect. Researchers studied the relationship between vitamin D daily doses and plasma 25(OH)D levels in the elderly or postmenopausal women with a maximum dose of 1000 or 1400 IU/day [6,7]. Heaney suggested that plasma 25(OH)D levels rise by 1 ng/mL (2.5 nmol/L) for every 100 IU (2500 ng) of daily vitamin D intake [8]. Where he and other researchers confirmed the linearity in the relationship between vitamin D supplementation and 25(OH)D in other populations [3,9], others reported a non-linear relationship for doses exceeding 1600–4000 IU per day [10,11,12]. In addition to daily supplementation, other supplementation frequencies (*i.e*., variable times per week or weekly, monthly or annually) were tested in various trials of varying periods of time [3,13,14]. Recent studies focused on the long half-life of vitamin D and investigated the long-term effects of supplementation [7,15]. However, these studies have not investigated the importance of dose, frequency and duration of supplementation for plasma 25(OH)D.

The objective of the present study is to examine the independent associations of dose, frequency and duration of vitamin D supplementation to plasma 25(OH)D in a large population-based sample of healthy participants.

## 2. Experimental Section

The Pure North S’Energy Foundation, a not-for-profit charitable organization, has provided free health and wellness services for volunteers since October 2007 [16]. Details of the program and data collection protocol are described elsewhere [16]. In brief, volunteer participants, mostly residing in the Canadian province of Alberta, were offered health promotion counseling and nutritional supplementation, with a particular focus on vitamin D given the Northern latitude of the locations where the program was offered. Participants were invited to complete a lifestyle questionnaire, have their heights and weights measured, and have blood drawn for the assessment of plasma 25(OH)D [17], among others [16].

Questionnaires included questions on dose, frequency, and duration of vitamin D supplementation. The dose was recorded as 1000–2000 IU, 2000–3000 IU, 3000–4000 IU and more than 5000 IU. Frequency of supplementation was recorded as never, one or two times, three to four times, five to six times and seven times per week, and duration of supplementation as one, two, three, four or five (or more) months. In the present study, we used 1000–2000 IU, once or twice per week, for one month as the minimum regimen for comparisons with higher doses, frequencies and durations, as well as for comparisons with those not taking vitamin D supplements.

The questionnaire included questions on physical activity, diet, smoking, season and place of residence. Physical activity was categorized as low, moderate and high [18]. Participants were asked to rate their general health as excellent, very good, good, fair, or needs improvement. As self-selection in the participation in the health and wellness program could be motivated by participants’ health status [19], and the program could be particularly attractive to those with relatively poor health, we also considered individuals’ health status at first visits in our analyses.

The Foundation anonymised their data prior to forwarding it to the University of Alberta for analyses. The present study made use of 4224 records of 2714 participants that completed the questionnaire and had plasma 25(OH)D measured between October 2007 and April 2012. One participant was excluded due to advanced kidney disease (estimated glomerular filtration rate <15 mL/min/1.73 m^2^), which affects the metabolism of vitamin D and the production of its active metabolites [20].

Plasma 25(OH)D values constituted the outcome in multilevel regressions models that tested the importance of dose, frequency and duration of vitamin D supplementation. Dose, frequency and duration were first analyzed univariably and subsequently multivariably to adjust for the potential confounders listed above. We applied multilevel regression to accommodate the hierarchical structure of the data, as records from one, two or more study visits were nested within observations of participants. Participants’ characteristics were considered at level 1 and visit specific characteristics at level 2. The regression analyses of the contribution of dose, frequency and duration to raising 25(OH)D were first analyzed without consideration of potential confounders (univariable) and subsequently with consideration (multivariable) of potential confounding of age, gender, body weight status, general health, season, physical activity, smoking status, and the consumption of milk (daily servings), fish (weekly servings) and margarine (frequency). To allow for our regression analyses to simultaneously analyze the associations of (1) no supplementation and (2) varying levels of supplementation in relation to 25(OH)D, we introduced interaction terms of (1) supplementation (Yes/No) and (2) dose, frequency and duration of supplementation. The resulting regression coefficients were to be interpreted as the changes in plasma 25(OH)D associated with (1) a minimum regimen relative to no supplementation and (2) a one-unit change in dose, frequency and duration of vitamin D supplementation relative to the minimum regimen. All analyses were conducted using STATA version 12 (College Station, TX, USA). Statistical significance was defined as p values less than 0.05 (two tails).

The Human Research Ethics Board of the University of Alberta had approved access to and analysis of the Foundation’s data for the purpose of the present analyses.

## 3. Results

From 2007 to 2012, there were 4224 visits made by 2714 participants. The mean age at visits was 42.48 ± 11.00 (mean ± SD, range = 9–85) years and the latitudes of their residence were 53.36 ± 2.49 (range = 42.93–68.30). The mean plasma 25(OH)D level was 96.41 ± 47.17 (range = 10–522) and 2068 visits (48.96%) were made between April and October. At visits with reported use of vitamin D supplementation, plasma 25(OH)D levels were, on average, 103.31 ± 47.58 nmol/L (range = 10–522), which is statistically significant more than 64.92 ± 28.93 nmol/L (range = 13.1–211) at visits without reported vitamin D supplementation (Table 1). Relative to visits without reporting supplementation use, visits with reported supplementations were more likely to be made by older participants, by those residing at lower latitudes, by overweight and obese participants, and by those reporting better health (*p* < 0.05 for all) (Table 1).

**Table 1:** Characteristics of 4224 visits by 2714 participants according to vitamin D supplementation use.

		Not taking vitamin D supplements (*N* = 759)			Taking any vitamin D supplements (*N* = 3465)			*P*
		Mean	SD		Mean	SD		
**25-hydroxyvitamin D (nmol/L)**		64.92	28.93		103.31	47.58		<0.001
**Age (years) **		35.78	11.03		42.48	11		<0.001
**Male (%)**		68.25%			69.99%			0.35
**Latitude (degrees) **		53.8	2.91		53.36	2.49		<0.001
**Body weight categories (%)**								<0.01
	Underweight	1.19%			0.52%			
	Normal weight	28.59%			24.01%			
	Overweight	36.50%			37.09%			
	Obesity	33.73%			38.38%			
**General Health (first visits) (%)**								<0.01
	Excellent	4.87%			6.58%			
	Very good	23.98%			0.2771			
	Good	41.90%			42.60%			
	Fair	17.13%			14.31%			
	Needs improvement	12.12%			8.80%			
**April to October (%)**		49.28%			48.89%			0.85
**Health behaviors**								
**Physical activity level (%)**								<0.01
	Low	37.88%			33.58%			
	Moderate	43.29%			51.87%			
	High	18.82%			14.56%			
**Smoking (%)**		16.16%			15.48%			0.64
**Food servings **								
**Milk (per day) (%)**								0.49
	**0**	6.18%			6.82%			
	**1 to 2**	64.61%			66.69%			
	**3 or more**	29.22%			26.49%			
**Fish (per week) (%)**								<0.001
	**0**	28.25%			19.17%			
	**1 to 2**	50.28%			59.11%			
	**3 or more**	21.47%			21.72%			
**Margarine consumption frequency (%)**								0.11
	Never	24.04%			27.99%			
	Rarely	34.13%			32.16%			
	Often	34.38%			30.28%			
	Always	7.45%			9.57%			

Note: 25(OH)D: plasma 25-hydroxyvitamin D levels. Categorical and continuous variables were tested with chi-square and two-sample t tests, respectively.

At those visits that participants reported to be taking vitamin D supplements, Table 2 shows the average plasma 25(OH)D levels by dose, frequency and duration of supplementation. The incremental increases of plasma 25(OH)D levels in each of these three dimensions were statistically significant (*p* < 0.001 for each of dose, frequency and duration). Compared to those using supplements one or two times per week (75.15 ± 31.86 nmol/L), those taking vitamin D supplements everyday had significantly higher 25(OH)D (129.37 ± 53.20 nmol/L, *p* < 0.001). Similarly, participants taking dosages of 5000 or more IU at a time (129.63 ± 55.88 nmol/L) had significantly higher 25(OH)D than those taking 1000–2000 IU each time (82.99 ± 34.77 nmol/L, *p* < 0.001). The average 25(OH)D for those taking vitamin D for five or more months (113.96 ± 48.89 nmol/L) was also significantly higher than for those only using vitamin D supplements for one month (83.35 ± 41.08 nmol/L, *p* < 0.001). The mean 25(OH)D levels associated with the highest doses, most frequent use and longest time of supplementation were significantly higher than those associated with the least intense supplementation regimens.

**Table 2:** Plasma 25-hydroxyvitamin D [25(OH)D] levels by dose, frequency and duration of vitamin D supplementation of 4224 study visits by 2714 participants.

		Not taking vitamin D supplementation			Taking any supplements			*P*
		No. of obs.	Mean	SD	No. of obs.	Mean	SD	
		759	64.76	28.86	3465	103.31	47.58	<0.001 *
**Dose (IU)**								
	1000 to 2000				1433	82.99	34.77	<0.001 **
	2000 to 3000				524	98.89	36.53	
	3000 to 4000				432	110.56	39.93	
	5000 or more				1076	129.63	55.88	
**Frequency, times/week**								
	1–2/week				738	75.15	31.86	<0.001 **
	3–4/week				762	89.28	37.94	
	5–6/week				935	108.27	41.76	
	7/week				1030	129.37	53.2	
**Duration (months)**								
	1				629	83.35	41.08	<0.001 **
	2				258	85.72	36.99	
	3				281	90.84	40.63	
	4				225	96.8	42.23	
	5 or more				2072	113.96	48.89	

Note: IU: international units; * Two-sample *t* test, comparing the mean values between those taking supplements or not; ** One-way ANOVA (analysis of variance) for different doses or frequencies or durations among those taking any supplements.

**Figure 1:**
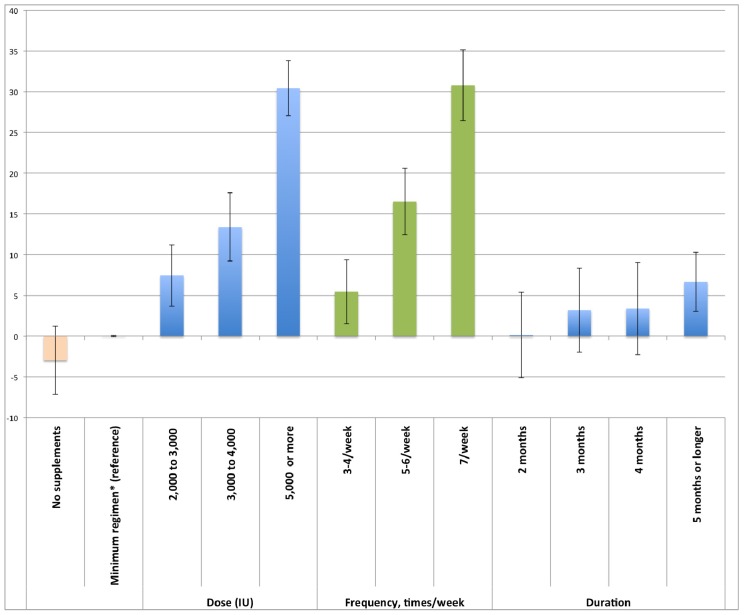
The dose responses between 25-hydroxyvitamin D levels and vitamin D supplementation based on the estimates from the multivariable regression model.

Note: All estimated changes in plasma 25-hydroxyvitamin D were compared to the minimum regimen* (1000–2000 IU once per week for one month). The error bars indicate the range of 95% confidence intervals. All estimates were adjusted for gender, age, body weight status, general health at first visits, latitudes, summer months (April to October), physical activity levels, smoking, and diet (milk and fish servings and frequency of margarine use).

Table 3 shows univariable and multivariable analysis of the association of dose, frequency and duration and other factors with plasma 25(OH)D levels. The multivariable analysis revealed that plasma 25(OH)D levels associated with minimum supplementation regimen (1000–2000 IU once or twice per week for a duration of one month) was not statistically significant higher than that when not taking supplements (β = 3.08; 95% CI: −1.10–7.26). Dose, frequency and duration of supplementation were each significantly associated with 25(OH)D levels (Table 3). Relative to the minimum regimen dose, those taking 2000–3000 IU was associated with 7.49 nmol/L (95% CI: 3.73–11.24) higher plasma 25(OH)D levels and higher doses were associated with higher plasma levels (Table 3). With respect to frequency, taking supplements 3–4 times per week was associated with 5.44 nmol/L (95% CI: 1.52–9.35) higher plasma levels compared to the minimum regimen. Any further increases in frequency, 5–6 times or 7 times per week, were associated with higher increase in plasma 25(OH)D levels. For those who had been supplementing vitamin D for five months or more, there were statistically significant increases of 6.68 nmol/L (95% CI: 3.05–10.31) in plasma 25(OH)D levels relative to those taking the minimum regimen. Figure 1 visualizes the dose response relationship of vitamin D supplementation and plasma 25(OH)D based on the figures presented in Table 3. The first bar drawn on the left shows the plasma 25(OH)D for those not taking supplements, compared to the plasma 25(OH)D level for a minimum regimen (1000–2000 IU once or twice per week for one month). The other bars visualize the estimated increase in plasma 25(OH)D resulting from doses, frequencies and durations exceeding those of the minimum regimen.

**Table 3:** The effects of dose, frequency and duration of vitamin D supplementation and individual characteristics on plasma 25(OH)D levels (nmol/L) among participants of a health and wellness program.

Models	Univariable analysis			Multivariable analysis		
	β	(95% CI)		β	(95% CI)	
**Vitamin D supplementation**						
**Taking minimum supplementation (compared to not taking any supplements)**	36.84 ***	(33.39 to	40.29)	3.08	(−1.10 to	7.26)
**Dose (IU)**						
**1000 to 2000**	(minimum regimen as reference)			(minimum regimen as reference)		
**2000 to 3000**	14.27 ***	(10.47 to	18.08)	7.49 ***	(3.73 to	11.24)
**3000 to 4000**	26.56 ***	(22.44 to	30.68)	13.19 ***	(9.02 to	17.36)
**5000 or more**	47.33 ***	(44.25 to	50.41)	30.22 ***	(26.86 to	33.59)
**Dose frequency**						
**1–2/week**	(minimum regimen as reference)			(minimum regimen as reference)		
**3–4/week**	14.09 ***	(10.07 to	18.10)	5.44 **	(1.52 to	9.35)
**5–6/week**	33.05 ***	(29.13 to	36.97)	16.52 ***	(12.41 to	20.64)
**7/week**	53.02 ***	(49.09 to	56.95)	30.69 ***	(26.35 to	35.03)
**Duration (months)**						
**1**	(minimum regimen as reference)			(minimum regimen as reference)		
**2**	2.87	(−3.11 to	8.85)	0.18	(−5.07 to	5.44)
**3**	6.61 *	(0.82 to	12.39)	3.22	(−1.94 to	8.38)
**4**	11.10 ***	(4.86 to	17.35)	3.31	(−2.34 to	8.97)
**5 or more**	28.56 ***	(24.82 to	32.30)	6.68 ***	(3.05 to	10.31)
**Age (per 10 years)**	7.65 ***	(6.35 to	8.96)	1.46 *	(0.28 to	2.63)
**Male (female as reference)**	−6.79 ***	(−10.19 to	−3.38)	−1.23	(−4.10 to	1.64)
**Body weight status**						
**Underweight**	−10.27	(−28.06 to	7.52)	−3.45	(−17.87 to	10.96)
**Normal**	(reference)			(reference)		
**Overweight**	−6.56 **	(−10.36 to	−2.76)	−7.13 ***	(−10.33 to	−3.93)
**Obesity**	−14.85 ***	(−18.71 to	−11.00)	−14.59 ***	(−17.97 to	−11.21)
**General health (first visit)**						
**Excellent**	(reference)			(reference)		
**Very good**	−11.01 **	(−17.89 to	−4.14)	−4.29	(−9.94 to	1.36)
**Good**	−19.08 ***	(−25.70 to	−12.46)	−7.98 **	(−13.52 to	−2.44)
**Fair**	−27.34 ***	(−34.73 to	−19.96)	−12.92 ***	(−19.15 to	−6.69)
**Needs improvement**	−30.20 ***	(−38.11 to	−22.29)	−11.24 **	(−17.97 to	−4.51)
**Latitude (per 10°)**	−17.50 ***	(−23.37 to	−11.63)	−5.39 *	(−10.17 to	−0.61)
**Summer months (April to October)**	1.18	(−1.50 to	3.86)	2.91 **	(0.74 to	5.09)
**Physical activity levels**						
**Low**	(reference)			(reference)		
**Moderate**	6.05 **	(2.58 to	9.52)	1.96 *	(−1.01 to	4.92)
**High**	9.99 ***	(5.06 to	14.93)	4.36	(0.04 to	8.68)
**Smoking**						
**Non-smoker**	(reference)			(reference)		
**Smoker**	−0.91	(−4.79 to	2.97)	−5.52 **	(−8.88 to	−2.17)
**Milk servings/day**						
**0**	(reference)			(reference)		
**1 to 2**	−5.14	(−11.69 to	1.41)	−3.92	(−9.46 to	1.62)
**3 or more**	−2.56	(−9.58 to	4.46)	−1.76	(−7.72 to	4.19)
**Fish servings/week**						
**0**	(reference)			(reference)		
**1 to 2**	0.33 *	(9.45 to	2.10)	−0.51	(−4.31 to	3.30)
**3 or more**	2.52 **	(13.51 to	2.86)	2.59	(−2.00 to	7.18)
**Margarine consumption frequency**						
**Never**	(reference)			(reference)		
**Rarely**	−6.12 **	(−10.33 to	−1.91)	−2.22	(−5.78 to	1.34)
**Often**	−12.89 ***	(−17.19 to	−8.59)	−4.75 *	(−8.43 to	−1.08)
**Always**	−7.60 *	(−13.74 to	−1.47)	−2.74	(−7.96 to	2.47)
**Constant**	(not shown)			114.46 ***	(87.25 to	141.68)
**No. of visits**	4224			4224		
**No. of individuals**	2714			2714		

Note: β = regression coefficients representing changes in plasma 25-hydroxyvitamin D (25(OH)D) (nmol/L). Minimum regimen: 1000**–**2000 IU once or twice per week for one month. * *p* < 0.05; ** *p* < 0.01; *** *p* < 0.001. The univariable analyses considered each covariate in separate analyses with the exception of dose, frequency and duration that were considered simultaneous. The constants of these univariable models were not shown. The multivariable analysis considered all variables included in the table as these have been reported to affect plasma 25-hydroxyvitamin D levels in the literature.

Age, summer season (April to October), and high levels of physical activity were associated in a statistically significant manner with higher plasma 25(OH)D levels (*p* < 0.05 for all). Males, northern latitudes, overweight or obesity, poor self-reported health and margarine intake were associated with lower 25(OH)D levels (*p* < 0.05 for all).

## 4. Discussion

We showed that participants with a minimum regimen (1000–2000 IU of vitamin D once or twice per week for one month) had plasma 25(OH)D levels that were not statistically higher than those of participants not taking supplements. This minimum regimen (1000–2000 IU, one or two times a week) is quantitatively similar to a daily intake of 143–571 IU. Clinical trials have tested compatible doses of 400 IU daily and showed benefits to bone density, but equivalent doses are not sufficient to prevent hip fractures or colorectal cancer [21,22]. To achieve the latter, individuals need higher plasma 25(OH)D levels, usually more than 75 nmol/L [23,24]. The effectiveness of the minimum regimen on improving plasma 25(OH)D levels is consistent with biological evidence. Lips suggested that catabolism rates of vitamin D are lower when plasma 25(OH)D levels are low [25]. This increases the propensity of accumulation at lower dosages.

We quantified the associations of dose, frequency and duration with plasma 25(OH)D and showed that the effects of dose and frequency were more pronounced than the effect of duration. We showed that only extending the supplementation duration to three or five months (or more) was correlated with substantial increases in plasma 25(OH)D. Whole-body distribution [26], half-life time [4], and plateau effects [12,23] may be the biological mechanisms underlying the modest contribution of regimens with duration of less than five months.

The dosing regimen promoted by the IOM is 600 IU for males and females between the ages of 1–70 years [2]. Others have advocated higher vitamin D intake [4,27]. The IOM recommendations are uniform and not distinct for individuals of varying body weight, sun exposure, health status and life style (physical activity, smoking status and diet). The present study revealed substantial contributions of these factors such that we believe they should be taken into consideration when choosing supplementation dosages to reach a target plasma 25(OH)D level.

The effects of body weight, sun exposure, and lifestyle on 25(OH)D[4] are also important to the design of clinical trials. Clinical trials testing varying vitamin D regimens may successfully control for heterogeneity between groups by random assignment, but may fail to adjust for the heterogeneity within groups [3]. To improve clinical trials of vitamin D regimens, we recommend that consideration be given not only to supplementation dose and frequency, but also to the individual characteristics. Examples include sun exposure advisories and season specific sun exposure advisories to trial participants, quantification of dietary intake, and an analytic approach that addresses body weight status.

We had observed that a minimum regimen (1000–2000 IU, one or two times a week, quantitatively similar to a daily intake of 143–571 IU) did not result in a substantial or statistically significant increase relative to not taking supplements (see Supplemental Table 1 to compare the results from previous studies). The Women’s Health Initiative used a regimen of 400 IU daily (which is in the range of our minimum regimen) and did not observe a reduction in all cardiometabolic outcomes examined [28,29]. Pittas *et al.* and Scragg [28,29] reported that 400 IU daily may have been too low a dose to achieve these benefits, which seem consistent with our observation of an insignificant effect of the minimum regimen.

The population-based approach with the consideration of various confounding factors and a large sample size should be considered as strengths of the present study [8,30]. As this study was conducted among healthy individuals, the results are generalizable to healthy individuals who seek to improve their vitamin D status. Other studies and trials had targeted populations with very specific characteristics, such as postmenopausal women [31] or the elderly only [32], or a small number of healthy men [9]. The adjustment of a number of confounding factors, such as obesity and physical activity, also makes the findings relevant to a broader population. However, we acknowledge that there are other existing confounding factors not taken into consideration. This study evaluated participants of a health and wellness program that is mostly offered to active workers. For this reason, socioeconomic status was considered to be homogenous. No questions related to income, education, job status, race and ethnicity, or family composition was included in the program’s questionnaires, which had been associated with 25(OH)D levels [33,34,35]. Limitations include the self-reported nature and reporting imprecision of some of the factors studied including dose, frequency and duration. Lastly, in the absence of better information on sun exposure, we used latitude and season as proxies for sunlight exposure, which may have introduced error and bias.

## 5. Conclusions

Each of the three dimensions of vitamin D supplementation—dose, frequency and duration—are independently associated with plasma 25(OH)D levels. The multivariable analysis reveals that plasma 25(OH)D levels associated with a minimum supplementation regimen (similar to a daily intake of 143–571 IU are not significantly higher than not taking supplements. These characteristics of the dose response relationship are not only useful for individuals to optimize plasma 25(OH)D, but also important for researchers to consider that, besides the assigned doses, there are other factors that influence plasma 25(OH)D levels and these should be adjusted for.

## Supplementary Files

Supplementary File 1 Supplementary Information (DOCX, 63 KB)
