# Supplementary material for: The Importance of Dose, Frequency and Duration of Vitamin D Supplementation for Plasma 25-Hydroxyvitamin D
Source: Nutrients. 2013 Oct 11;5(10):4067–78. doi: 10.3390/nu5104067 (PMC3820059; doi:10.3390/nu5104067)
Supplement: Supplementary File 1 — Supplementary Information (DOCX, 63 KB) [file nutrients-05-04067-s001.docx]

**Supplementary Information**

**Table S1.** Studies that reported on intervention doses less than 600 IU daily in relation to changes in plasma 25(OH)D levels.

| Author  (publication year) | *N* | Population characteristics | Daily vitamin D dose (IU) | Isoform of vitamin D supplements | Baseline 25(OH)D (nmol/L) | End of study 25(OH)D (nmol/L) | Absolute (%) change in 25(OH)D (nmol/L) |
| --- | --- | --- | --- | --- | --- | --- | --- |
| Keane (1998) [[1](#_ENREF_1)] | 23 | community-based,  elderly subjects | 200 | Unspecified | 24 * | 46.25 * | 22.3 (92.9) |
| Lau (2001) [[2](#_ENREF_2)] | 95 | postmenopausal women | 240 | D_3_ | 66 | 89.2 | 23.2 (35.1) |
| McKenna (1995) [[3](#_ENREF_3)] | 28 | healthy adults | 137 | D_3_ | 77 | 62 | −15 (−19.5) |
| Palacios (2005) [[4](#_ENREF_4)] | 34 | postmenopausal women | 228 | D_3_ | 109.9 * | 123.9 * | 14 (12.7) |
| Chee (2003) [[5](#_ENREF_5)] | 91 | postmenopausal women | 400 | D_3_ | 69.1 | 86.4 | 17.2 (25.0) |
| de Jong (1999) [[6](#_ENREF_6)] | 76 | free-living frail  elderly people | 400 | Unspecified | 37 | 72 | 35 (94.6) |
| Natri (2006) [[7](#_ENREF_7)] | 21 | healthy women | 400 | D_3_ | 29 | 45.3 | 16.3 (56.2) |
| Panunzio (2003) [[8](#_ENREF_8)] | 98 | healthy adults | 400 | Unspecified | 40.2 * | 81.3 * | 41.1 (102.2) |

Note: Only studies with doses compatible with the minimum regimen of this study [1000 or 2000 IU once or twice per week; 142.8 IU daily (1000/7) to 571.4 IU daily (2000 × 2/7)] were included in this table. *N*: number of observations; * 25(OH)D_3_; IU, international units.

**Table S2.** Assessment of the independence of vitamin D supplementation dose, frequency and duration in relation to plasma 25(OH)D levels (nmol/L).

| Vitamin D supplementation | | |  | | | Coef. | (95% CI) |  | P |
| --- | --- | --- | --- | --- | --- | --- | --- | --- | --- |
| Taking baseline supplementation *versus* not taking any supplements | | | | | | **(not used to assess  interaction terms)** | | |  |
| Dose (IU) | | |  | |  |  |  |  |  |
|  | 1000–2000 IU | | |  | | (minimum regimen as reference) | | | |
|  | 2–3000 IU |  | |  | | 14.32 | (−1.00 to | 29.65), | *p* = 0.07 |
|  | 3–4000 IU |  | |  | | 11.23 | (−7.28 to | 29.74), | *p* = 0.24 |
|  | 5000 + IU |  | |  | | 10.43 | (−1.89 to | 22.75), | *p* = 0.10 |
| Dose frequency | | |  | |  |  |  |  |  |
|  | 1–2/week |  | |  | | (minimum regimen as reference) | | | |
|  | 3–4/week |  | |  | | 4.14 | (−3.59 to | 11.87), | *p* = 0.29 |
|  | 5–6/week |  | |  | | 18.36 | (7.95 to | 28.77), | *p* < 0.01 |
|  | 7/week |  | |  | | 16.28 | (4.94 to | 27.63), | *p* = 0.01 |
| Duration | | |  | |  |  |  |  |  |
|  | 1 month |  | |  | | (minimum regimen as reference) | | | |
|  | 2 months |  | |  | | 2.67 | (−5.98 to | 11.33), | *p* = 0.55 |
|  | 3 months |  | |  | | 7.9 | (−1.95 to | 17.76), | *p* = 0.12 |
|  | 4 months |  | |  | | 2.07 | (−12.99 to | 17.12), | *p* = 0.79 |
|  | 5 + months |  | |  | | 4.17 | (−2.66 to | 11.00), | *p* = 0.23 |
| Age (per 10 years) | | |  | |  | 1.43 | (0.07 to | 2.80), | *p* = 0.04 |
| Male (female as reference) | | | |  | | 1.1 | (−2.22 to | 4.41), | *p* = 0.52 |
| Latitude (per 10°) | | |  | |  | −5.92 | (−11.61 to | −0.24), | *p* = 0.04 |
| Body weight status | | |  | |  |  |  |  |  |
|  | Underweight | | |  | | −1.39 | (−19.77 to | 16.99), | *p* = 0.88 |
|  | Normal |  | |  | | (reference) | | |  |
|  | Overweight |  | |  | | −7.75 | (−11.45 to | −4.05), | *p* < 0.01 |
|  | Obesity |  | |  | | −16.72 | (−20.61 to | −12.83), | *p* < 0.01 |
| General health (first visit) | | | |  | |  |  |  |  |
|  | Excellent |  | |  | | (reference) | | |  |

**Table S2.** *Cont.*

|  | Very good |  | |  | | −5.46 | (−11.81 to | 0.90), | *p* = 0.09 |
| --- | --- | --- | --- | --- | --- | --- | --- | --- | --- |
|  | Good |  | |  | | −9.1 | (−15.34 to | −2.87), | *p* < 0.01 |
|  | Fair |  | |  | | −14.18 | (−21.27 to | −7.09), | *p* < 0.01 |
|  | Needs improvement | | |  | | −11.5 | (−19.21 to | −3.79), | *p* < 0.01 |
| Summer months (April to October) | | | |  | | 1.35 | (−1.12 to | 3.81), | *p* = 0.29 |
| Physical activity levels | | |  | |  |  |  |  |  |
|  | Low |  | |  | | (reference) | | |  |
|  | Moderate |  | |  | | 1.46 | (−1.86 to | 4.78), | *p* = 0.39 |
|  | High |  | |  | | 4.18 | (−0.72 to | 9.09), | *p* = 0.10 |
| Smoking | | |  | |  |  |  |  |  |
|  | Non-smoker |  | |  | | (reference) | | |  |
|  | Smoker |  | |  | | −6.36 | (−10.19 to | −2.52), | *p* < 0.01 |
| Diet and nutrition | | |  | |  |  |  |  |  |
| Milk servings/day | | |  | |  |  |  |  |  |
|  | 0 |  | |  | | (reference) | | |  |
|  | 1 to 2 |  | |  | | −5 | (−11.17 to | 1.17), | *p* = 0.11 |
|  | 3 or more |  | |  | | −1.7 | (−8.37 to | 4.97), | *p* = 0.62 |
| Taking baseline supplementation *versus* not taking any supplements | | | | | | **(not used to assess  interaction terms)** | | |  |
| Fish servings/week | | |  | |  |  |  |  |  |
|  | 0 |  | |  | | (reference) | | |  |
|  | 1 to 2 |  | |  | | −2 | (−6.37 to | 2.36), | *p* = 0.37 |
|  | 3 or more |  | |  | | 2.28 | (−2.96 to | 7.51), | *p* = 0.39 |
| Margarine consumption frequency | | | |  | |  |  |  |  |
|  | Never |  | |  | | (reference) | | |  |
|  | Rarely |  | |  | | −3.35 | (−7.32 to | 0.63), | *p* = 0.10 |
|  | Often |  | |  | | −5.22 | (−9.35 to | −1.10), | *p* = 0.01 |
|  | Always |  | |  | | −2.83 | (−8.62 to | 2.96), | *p* = 0.34 |
| Frequency*Dose | | |  | |  |  |  |  |  |
| 1–2/week | 1000–2000 IU | | |  | | (used as the minimum regimen) | | |  |
| 3–4/week | 2–3000 IU |  | |  | | 0.92 | (−12.52 to | 14.37), | *p* = 0.89 |
| 3–4/week | 3–4000 IU |  | |  | | −7.35 | (−24.72 to | 10.02), | *p* = 0.41 |
| 3–4/week | 5000 + IU |  | |  | | 0.87 | (−10.97 to | 12.71), | *p* = 0.89 |
| 5–6/week | 1000–2000 IU | | |  | | (equivalent to one of the main effects) | | | |
| 5–6/week | 2–3000 IU |  | |  | | 5.67 | (−8.47 to | 19.81), | *p* = 0.43 |
| 5–6/week | 3–4000 IU |  | |  | | −6.4 | (−22.94 to | 10.15), | *p* = 0.45 |
| 5–6/week | 5000 + IU |  | |  | | 14.67 | (3.34 to | 26.01), | *p* = 0.01 |
| 7/week | 1000–2000 IU | | |  | | (equivalent to one of the main effects) | | | |
| 7/week | 2–3000 IU |  | |  | | 8.34 | (−6.10 to | 22.79), | *p* = 0.26 |
| 7/week | 3–4000 IU |  | |  | | 3.77 | (−13.76 to | 21.30), | *p* = 0.67 |
| 7/week | 5000 + IU |  | |  | | 30.34 | (19.03 to | 41.64), | *p* < 0.01 |
| Dose*Duration | | |  | |  |  | | |  |
| 1000–2000 IU | 1 month |  | |  | | (used as the minimum regimen) | | |  |
| 2000–3000 IU | 2 months |  | |  | | −14.79 | (−32.53 to | 2.96), | *p* = 0.10 |
| 2000–3000 IU | 3 months |  | |  | | −8.7 | (−25.49 to | 8.10), | *p* = 0.31 |
| 2000–3000 IU | 4 months |  | |  | | −23.12 | (−40.82 to | −5.43), | *p* = 0.01 |
| 2000–3000 IU | 5+ months |  | |  | | −9.27 | (−22.70 to | 4.16), | *p* = 0.18 |
| 3000–4000 IU | 1 month |  | |  | | (equivalent to one of the main effects) | | | |
| 3000–4000 IU | 2 months |  | |  | | −6.8 | (−30.16 to | 16.57), | *p* = 0.57 |
| 3000–4000 IU | 3 months |  | |  | | −7 | (−26.15 to | 12.16), | *p* = 0.47 |

**Table S2.** *Cont.*

| 3000–4000 IU | 4 months |  | |  | | 16.13 | (−3.88 to | 36.14), | *p* = 0.11 |
| --- | --- | --- | --- | --- | --- | --- | --- | --- | --- |
| 3000–4000 IU | 5+ months |  | |  | | 8.4 | (−6.16 to | 22.97), | *p* = 0.26 |
| 5000+ IU | 1 month |  | |  | | (equivalent to one of the main effects) | | | |
| 5000+ IU | 2 months |  | |  | | 3 | (−13.29 to | 19.30), | *p* = 0.72 |
| 5000+ IU | 3 months |  | |  | | −9.29 | (−25.26 to | 6.68), | *p* = 0.25 |
| 5000+ IU | 4 months |  | |  | | −2.56 | (−19.77 to | 14.66), | *p* = 0.77 |
| 5000+ IU | 5+ months |  | |  | | 4.94 | (−5.41 to | 15.30), | *p* = 0.35 |
| Frequency*Duration | | |  | |  |  | | |  |
| 1–2/week | 1 month |  | |  | | (used as the minimum regimen) | | |  |
| 3–4/week | 2 months |  | |  | | 6.94 | (−7.10 to | 20.99), | *p* = 0.33 |
| 3–4/week | 3 months |  | |  | | 3.06 | (−10.82 to | 16.94), | *p* = 0.67 |
| 3–4/week | 4 months |  | |  | | 18.07 | (−0.72 to | 36.87), | *p* = 0.06 |
| 3–4/week | 5+ months |  | |  | | 3.45 | (−6.76 to | 13.66), | *p* = 0.51 |
| 5–6/week | 1 month |  | |  | | (equivalent to one of the main effects) | | | |
| Taking baseline supplementation versus not taking any supplements | | | | | | **(not used to assess  interaction terms)** | | |  |
| 5–6/week | 2 months |  | |  | | −5.26 | (−21.39 to | 10.87), | *p* = 0.52 |
| 5–6/week | 3 months |  | |  | | −9.21 | (−25.50 to | 7.09), | *p* = 0.27 |
| 5–6/week | 4 months |  | |  | | −2.06 | (−21.80 to | 17.67), | *p* = 0.84 |
| 5–6/week | 5 + months |  | |  | | −0.23 | (−11.84 to | 11.38), | *p* = 0.97 |
| 7/week | 1 month |  | |  | | (equivalent to one of the main effects) | | | |
| 7/week | 2 months |  | |  | | −11.96 | (−32.25 to | 8.34), | *p* = 0.25 |
| 7/week | 3 months |  | |  | | 6.85 | (−11.96 to | 25.65), | *p* = 0.48 |
| 7/week | 4 months |  | |  | | −0.3 | (−22.95 to | 22.35), | *p* = 0.98 |
| 7/week | 5+ months |  | |  | | 6.43 | (−5.73 to | 18.60), | *p* = 0.30 |
| Constant |  |  | |  | | 126.79 | (94.90 to | 158.68), | *p* < 0.01 |
|  |  |  | |  | |  |  |  |  |
| No. of visits |  |  | |  | | 3465 |  |  |  |
| No. of individuals | | |  | |  | 2188 |  |  |  |

Note: Only those reporting any use of vitamin D supplementation were eligible for this analysis.
25(OH)D = 25-hydroxyvitamin D.

References

1. Keane, E.M.; Healy, M.; O’Moore, R.; Coakley, D.; Walsh, J.B. Vitamin D-fortified liquid milk: Benefits for the elderly community-based population*. Calcif. Tissue Int*. **1998**, *62*, 300–302.
2. Lau, E.M.; Woo, J.; Lam, V.; Hong, A. Milk supplementation of the diet of postmenopausal Chinese women on a low calcium intake retards bone loss. *J. Bone Miner. Res*. **2001**, *16*,
   1704–1709.
3. McKenna, M.J.; Freaney, R.; Byrne, P.; McBrinn, Y.; Murray, B.; Kelly, M.; Donne, B.; O’Brien, M. Safety and efficacy of increasing wintertime vitamin D and calcium intake by milk fortification. *QJM* **1995**, *88*, 895–898.
4. Palacios, S.; Castelo-Branco, C.; Cifuentes, I.; von Helde, S.; Baro, L.; Tapia-Ruano, C.; Menendez, C.; Rueda, C. Changes in bone turnover markers after calcium-enriched milk supplementation in healthy postmenopausal women: A randomized, double-blind, prospective clinical trial. *Menopause* **2005**, *12*, 63–68.
5. Chee, W.S.; Suriah, A.R.; Chan, S.P.; Zaitun, Y.; Chan, Y.M. The effect of milk supplementation on bone mineral density in postmenopausal Chinese women in Malaysia. *Osteoporos Int.* **2003**, *14*, 828–834.
6. De Jong, N.; Chin, A.; Paw, M.J.; de Groot, L.C.; de Graaf, C.; Kok, F.J.; van Staveren, W.A. Functional biochemical and nutrient indices in frail elderly people are partly affected by dietary supplements but not by exercise. *J. Nutr*. **1999**, *129*, 2028–2036.
7. Natri, A.M.; Salo, P.; Vikstedt, T.; Palssa, A.; Huttunen, M.; Karkkainen, M.U.; Salovaara, H.; Piironen, V.; Jakobsen, J.; Lamberg-Allardt, C.J. Bread fortified with cholecalciferol increases the serum 25-hydroxyvitamin D concentration in women as effectively as a cholecalciferol supplement. *J. Nutr*. **2006**, *136*, 123–127.
8. Panunzio, M.F.; Pisano, A.; Telesforo, P.; Tomaiuolo, P. Diet can increase 25-hydroxyvitamin-D3 plasma levels in the elderly: A dietary intervention trial. *Nutr. Res.* **2003**, *23*, 1177–1181.
